# Diagnostic yield of urinary tract ultrasound in children following a first urinary tract infection: a retrospective cohort study

**DOI:** 10.1007/s00247-026-06729-5

**Published:** 2026-07-30

**Authors:** Tjaš Žvar, Peter Slak, Domen Plut

**Affiliations:** 1 Department of Radiology, Faculty of Medicine, University of Ljubljana, Vrazov trg 2, 1000 Ljubljana, Slovenia; 2 Division of Pediatric Radiology, Institute of Radiology, University Medical Centre Ljubljana, Zaloška cesta 2, 1000 Ljubljana, Slovenia

**Keywords:** Child, Congenital abnormalities, Contrast media, Pediatrics, Ultrasonography, Urinary tract infection

## Abstract

**Background:**

Urinary tract ultrasound (US) is widely recommended following a first urinary tract infection (UTI) in children to detect congenital anomalies of the kidney and urinary tract (CAKUT). However, its routine use has been questioned, given the limited diagnostic yield of US for certain anomalies.

**Objective:**

To assess the diagnostic yield of urinary tract US after a first UTI and its impact on clinical management.

**Materials and methods:**

This retrospective study included 1,460 children who underwent urinary tract US following a first UTI between January 2014 and December 2024. Children were stratified by age and classified into five diagnostic categories based on US findings, with follow-up data reviewed to assess final diagnoses.

**Results:**

Most children were classified with normal US (74.3%). CAKUT was identified in 6.9%, while CAKUT with dilation was detected in 3.5%. Vesicoureteral reflux (VUR) was the most common CAKUT with dilation (1.64%). Most children with VUR (89.7%) were ≤24 months old. CAKUT with dilation was more common in males (*P*=0.003) and infants aged 0–6 months (*P*=0.04), representing 35.6% of the study population. Despite a normal initial US, 34 additional cases of VUR were identified after recurrent UTI.

**Conclusion:**

Routine US after a first UTI in all children detects few clinically relevant abnormalities, does not reliably guide the need for reflux studies, and presents a burden for the health system; therefore, selective imaging strategies should be considered. Our findings support the consideration of a more selective imaging strategy. A two-tier imaging approach may be warranted for infants younger than 6 months and all male children following a first UTI, and children with recurrent UTIs.

**Graphical Abstract:**

**Figure:**
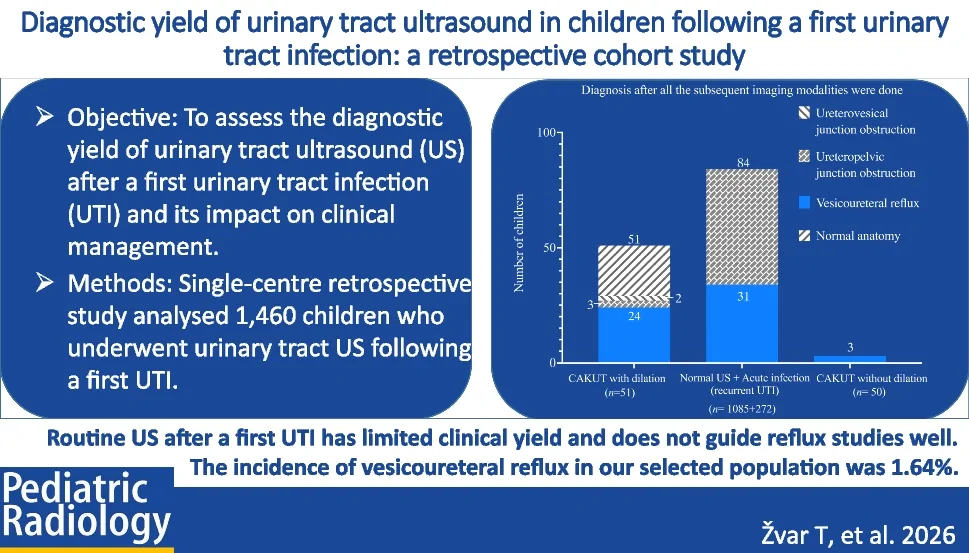

## Introduction

Urinary tract infections (UTIs) are one of the most common infections in early childhood [1]. Etiopathology of the disease is usually the ascending spread of bacterial colonies around the urethra. Less commonly, the infection is hematogenous [1]. Main risk factors for UTIs in children include female sex, neurogenic bladder defects, and congenital anomalies of the kidney and urinary tract (CAKUT) [1]. Urinary tract ultrasound (US) is a non-invasive and readily accessible radiologic imaging modality that provides detailed evaluation of the urinary system. It is commonly included in guidelines for the management of a child with a first UTI [2–7]. US plays a key role in identifying complicated infections (e.g., abscess, phlegmon) and early detection of CAKUT, which can predispose to recurrent or severe UTIs, renal cortical scarring, and ultimately chronic kidney disease (CKD) [1, 8–11]. The most common CAKUT is vesicoureteral reflux (VUR) in 25%, followed by ureteropelvic junction obstruction (UPJO) and ureterovesical junction obstruction (UVJO), each affecting 11% [11].

Although US is generally considered a patient- and system-friendly imaging modality, it nonetheless imposes a burden on both the patient and the healthcare system [12, 13]. Several concerns have been raised regarding the feasibility of performing urinary tract US in all children with UTIs [8]. Firstly, UTIs are highly prevalent in infants and toddlers, resulting in a large number of children undergoing US examinations [14, 15]. Secondly, the etiology of these infections is typically extrinsic, namely, the ascending spread of uropathogens, which is independent of the presence of CAKUT [2]. Thirdly, VUR, the most common form of CAKUT, cannot be reliably diagnosed using US, and physiological low-grade VUR is frequently observed during the first year of life [16, 17]. Finally, complicated UTIs are usually evident through a prolonged or more severe clinical course, allowing for clinical stratification in such cases [3].

To date, only a limited number of studies have investigated the diagnostic yield of urinary tract US in children at the time of first UTI [9, 10, 16, 18–20]. These studies often involved small cohorts, focused on acute findings or isolated forms of CAKUT, and lacked long-term follow-up. Our study aimed to evaluate the diagnostic yield of urinary tract US in children following their first UTI and to assess its impact on subsequent clinical management.

## Materials and methods

This retrospective study was approved by the National Medical Ethics Committee of the Republic of Slovenia. All data were obtained from the institutional electronic medical records system. The study population included all children referred to our institution for urinary tract US between January 2014 and December 2024, with a clinical indication of a first UTI.

The diagnosis of UTI was based on our national guidelines, which rely on clinical presentation with fever and laboratory results of blood and urine. The diagnosis is supported by leukocytosis with a left shift, elevated C-reactive protein and procalcitonin levels in blood, alongside bacteriuria, leukocyturia, and the presence of nitrites in the urine. For children up to 3 years of age, urine samples were collected using pediatric urine bags.

US was not performed at a uniform time point in relation to the UTI. Some children underwent the examination during the acute phase of the UTI, while in others the examination was performed shortly after it. Children with known CAKUT before the first UTI or a pathological neonatal US exam were excluded. All retrieved data were anonymized before analysis.

The following variables were collected: (1) sex, (2) date of birth, (3) date of US examination, (4) US findings, (5) follow-up data. Follow-up data included (a) diagnostic work-up and final diagnosis in children with clinically significant US findings and (b) review of the final medical record for all children to determine whether any CAKUT had been diagnosed later in life. The child’s age at the time of US examination was calculated based on the date of birth and the date of imaging.

For analysis, children were categorized into three age groups based on the National Institute for Health and Care Excellence guidelines [21]: (i) 0–6 months, (ii) 7–24 months, and (iii) >24 months. This age stratification reflects developmental and clinical differences in UTI presentation, diagnostic work-up thresholds, and the prevalence of CAKUT.

Based on US findings, children were further classified into five diagnostic categories: (a) normal US exam (“normal US”), (b) sonographic signs of acute infection without CAKUT (“acute infection”), (c) congenital anomalies of the kidney and urinary tract without dilation (“CAKUT without dilation”), (d) congenital anomalies of the kidney and urinary tract with dilation (“CAKUT with dilation”), and (e) other findings (“other”). The “normal US” category was defined as having a morphologically normal urinary tract on US. The “acute infection” category included children with sonographic signs suggestive of acute UTI, such as (i) pelvic or ureteral wall thickening, (ii) echogenic content or cellular debris within the urinary tract, (iii) blurred corticomedullary differentiation of the renal parenchyma, (iv) increased renal parenchymal echogenicity, or (v) mild renal enlargement [9, 22]. If a child demonstrated sonographic features of both acute UTI and CAKUT, they were classified into one of the CAKUT categories. The “CAKUT without dilation” category comprised children with findings such as simple renal cyst, complex renal cyst, a fibrotic urachal remnant, ureterocele, hypoplastic kidney, renal agenesis, and anatomical variants like a horseshoe kidney or a duplex collecting system (DCS) without associated urinary tract dilation. In contrast, the “CAKUT with dilation” category included children with anomalies that present on US with urinary tract dilation, such as VUR, UPJO, UVJO, and anatomical variants like a horseshoe kidney or a DCS with associated dilation. The “other” category encompassed clinically significant pathologic findings not related to CAKUT.

For children classified in the “CAKUT with dilation” category, follow-up data were reviewed to determine the final diagnosis. Notably, our institution serves as the sole national referral center for pediatric urinary tract surgical procedures. Consequently, in cases where no additional diagnostic evaluation (i.e., contrast-enhanced voiding urosonography (ceVUS), voiding cystourethrography (VCUG), or dynamic renal scintigraphy) was performed at our institution, children were presumed not to have a clinically significant dilation. This assumption is based on the rationale that children followed up at other institutions would likely have eventually been referred to our center for therapeutic intervention.

### Statistics

Categorical variables were described as absolute numbers and percentages. The chi-square test of independence was used to compare categorical variables across groups; Fisher’s exact test was applied when expected counts were <5. Proportions between two groups were compared using the two-proportion *z*-test. Sex distribution across age groups was analyzed with the chi-square goodness-of-fit test. To examine the effect of sex and age as variables for CAKUT with dilation, we used a multivariate binomial logistic regression model. Statistical analyses were performed with IBM SPSS Statistics, version 30.0 (IBM Corp., Armonk, NY). A *P*-value <0.05 was considered statistically significant.

## Results

### Demographic and population characteristics

**Fig. 1:**
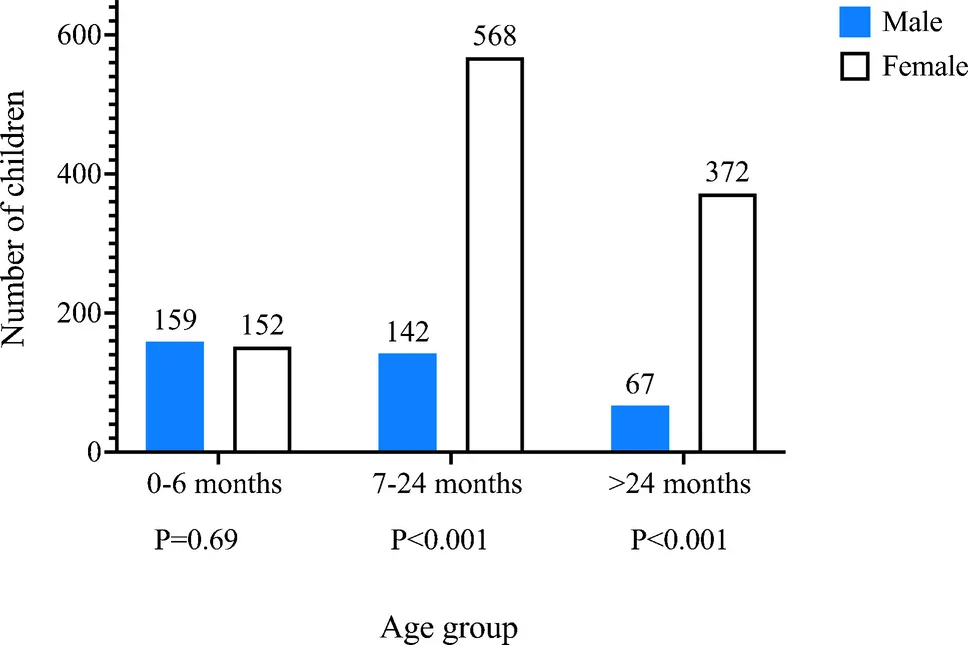
Sex distribution in three different age groups of children. *P*-values for group comparisons are displayed under each age group in the figure

The study included 1,460 children (25.2% male, 74.8% female). The age range was between 5–8,234 days, median 410 days. The cohort was divided into three age groups for the purpose of analysis. The sex distribution of children in all the groups is presented in Fig. 1.

### Urinary tract US findings

Table 1 provides a detailed breakdown of the US examination findings following a first-time UTI, including age and sex distribution for each category. Furthermore, Table 2 details the types and frequencies of CAKUT encompassed within these categories.

**Table 1:** Distribution of sex and age groups in different diagnostic categories based on urinary tract ultrasound

	Normal US *n*=1,085 (74.3)	Acute infection *n*=272 (18.6)	CAKUT without dilation *n*=50 (3.4)	CAKUT with dilation *n*=51 (3.5)	Other *n*=2 (0.1)
Sex *n* (%)					
Male (*n*=368)	263/368 (71.5)	69/368 (18.8)	14/368 (3.8)	22/368 (6.0)	0/368 (0.0)
Female (*n*=1,092)	822/1,092 (75.2)	203/1,092 (18.6)	36/1,092 (3.3)	29/1,092 (2.7)	2/1,092 (0.2)
* P*-value	0.16	0.94	0.65	**0.003**	1
Age group *n* (%)					
0–6 months (*n*=311)	222/311 (71.4)	59/311 (19.0)	12/311 (3.9)	18/311 (5.8)	0/311 (0.0)
7–24 months (*n*=710)	560/710 (78.9)	112/710 (15.8)	16/710 (2.3)	22/710 (3.1)	0/710 (0.0)
>24 months (*n*=439)	303/439 (69.0)	101/439 (23.0)	22/439 (5.0)	11/439 (2.5)	2/439 (0.5)
* P*-value	**<0.001**	**0.011**	0.078	**0.04**	0.21

“*n*” denotes the number of children, and numbers in parentheses represent the percentage of the total value in the row. Statistically significant differences are shown in bold

Abbreviations:* CAKUT* congenital anomalies of the kidney and urinary tract

**Table 2:** Distribution of congenital abnormalities of the urinary tract by age groups

Congenital abnormality *n* (%)	Age group			*P*-value
	0–6 months (*n*=311)	7–24 months (*n*=710)	>24 months (*n*=439)	
CAKUT with dilation				
Urinary tract dilation (*n*=50)	17/50 (34.0)	22/50 (44.0)	11/50 (22.0)	0.065
DCS with dilation (*n*=1)	1/1 (100.0)	0/1 (0.0)	0/1 (0.0)	/
CAKUT without dilation				
Simple cyst (*n*=8)	2/8 (25.0)	2/8 (25.0)	4/8 (50.0)	0.47
Hypoplastic kidney (*n*=1)	1/1 (100.0)	0/1 (0.0)	0/1 (0.0)	/
Renal agenesis (*n*=3)	1/3 (33.3)	0/3 (0.0)	2/3 (66.7)	0.49
DCS without dilation (*n*=22)	5/22 (22.7)	9/22 (40.9)	8/22 (36.4)	0.41
Horseshoe kidney (*n*=7)	2/7 (28.6)	2/7 (28.6)	3/7 (42.9)	0.84
Urachal anomaly (*n*=4)	0/4 (0.0)	0/4 (0.0)	4/4 (100.0)	**0.007**
Ureterocele (*n*=3)	1/3 (33.3)	2/3 (66.7)	0/3 (0.0)	0.45
Complex cyst (*n*=1)	0/1 (0.0)	1/1 (100.0)	0/1 (0.0)	/
Neurogenic bladder (*n*=1)	0/1 (0.0)	0/1 (0.0)	1/1 (100.0)	/
Other				
Nephrolithiasis (*n*=1)	0/1 (0.0)	0/1 (0.0)	1/1 (100.0)	/
Acute appendicitis (*n*=1)	0/1 (0.0)	0/1 (0.0)	1/1 (100.0)	/

“*n*” denotes the number of children, and numbers in parentheses represent the percentage of the total number in the row. Statistically significant differences are shown in bold

Abbreviations:* CAKUT* congenital anomalies of the kidney and urinary tract, *DCS* duplex collecting system

Incidence of CAKUT with dilation was more common in males (6%) and the age group 0–6 months (5.8%). These two groups combined 520 children (35.6%).

The multivariate binomial logistic regression model showed that sex was a statistically significant independent predictor (*P*=0.027), with males having nearly twice the odds for CAKUT with dilation compared to females (OR=1.981; 95% CI, 1.080–3.630). The age group variable was not of statistical significance (*P*=0.138 and *P*=0.632).

**Fig. 2:**
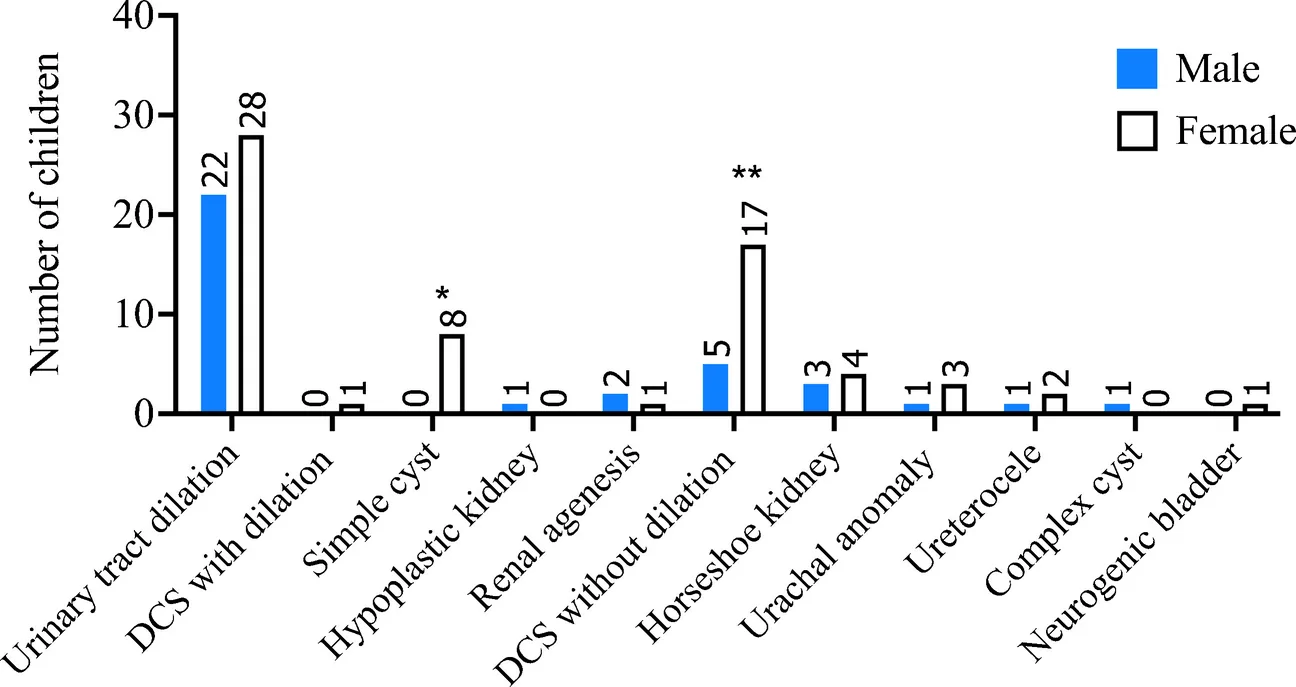
Distribution of sexes for individual congenital anomalies of the kidney and urinary tract diagnoses. Statistically significant differences are marked with an *asterisk* (*P=0.008 and **P=0.011). *DCS* duplex collecting system

A comparison of the distribution of individual diagnoses between sexes, assuming an even distribution, showed statistically significant differences in two diagnoses (Fig. 2). A simple cyst was observed exclusively in females, while the diagnosis of DCS without dilation was 3.4 times more common in females than in males.

### Follow-up

All children were followed up for at least a period of 1 year. All the children with “CAKUT with dilation” (*n*=51) underwent further diagnostics with ceVUS or dynamic renal scintigraphy. VUR was determined in 24 children (1.64% of the study group, 47.1% of the “CAKUT with dilation”), UPJO in three (0.2% of the study group, 5.9% of the “CAKUT with dilation”), and UVJO in two (0.1% of the study group, 3.9% of the “CAKUT with dilation”). Thirteen of the 24 children with VUR had bilateral VUR. In a single child, a ureterocele was diagnosed during the ceVUS examination. In 21 children, no CAKUT was diagnosed.

In two (66.7%) children with an initial diagnosis of ureterocele, the ureterocele spontaneously resolved on follow-up. Spontaneous resolution was also observed in three simple cysts, while one simple cyst was later confirmed as autosomal dominant polycistic kidney disease (ADPKD).

In the categories “normal US” (*n*=1,085) and “acute infection” (*n*=272), 84 children had recurrent UTIs that warranted additional evaluation with ceVUS. Of these 84 children, 31 (36.9%) were diagnosed with VUR, of which 14 had bilateral and 17 had one-sided VUR.

Follow-up in the category of children with “CAKUT without dilation” showed that three children had a positive ceVUS in addition to their original diagnosis: a child with a horseshoe kidney and two children with a DCS.

In this group of 34 children with VUR and normal initial US, the mean time between the initial US and VUR diagnosis (i.e., ceVUS) was 265 days (SD 451; minimum 16, maximum 1,770).

Ultimately, the study group included 58 children with confirmed VUR. Out of these children, 24 (41.4%) presented with urinary tract dilation at the time of first UTI. Fifty-two (89.7%) of these children were 24 months or less old.

## Discussion

Our study aimed to evaluate the diagnostic yield of urinary tract US in children following their first UTI and to determine its impact on subsequent clinical management. We found that US detected CAKUT in 6.9% (*n*=101) of these children. However, “CAKUT with dilation,” which is clinically more important, was detected only in 3.5% (*n*=51). VUR was the most commonly diagnosed CAKUT, present in 23.8% (*n*=24) of children with CAKUT, corresponding to just 1.64% of the overall study population. Notably, this rate appears comparable to the estimated prevalence of VUR in the general population (1–2%) [17, 23]. Taken together, these findings suggest that routine urinary tract US following the first UTI in children has a low diagnostic yield. Given the large number of children undergoing imaging to identify a very small proportion of clinically significant findings, this practice places a substantial burden on the healthcare system, consuming resources, increasing workload for medical staff, and generating additional appointments and anxiety for patients and their families. Our results call into question the routine use of US in this setting and support a more selective, risk-based approach.

CAKUT with dilation was significantly more common in males (6.0%; *P*=0.003) and infants aged 0–6 months (5.8%; *P*=0.04). In contrast, the incidence across all other demographic subgroups was low, ranging between 2.5% and 3.1%. Together, these two high-risk cohorts account for 35.6% of our total study population but represent the majority (62.7%) of CAKUT with dilation. These findings support transitioning toward a more selective imaging strategy. Specifically, a two-tier imaging approach may be warranted. Initially, targeting only male children and infants younger than 6 months, and secondly, children of any demographic with recurrent UTIs. Implementing this selective protocol could achieve a 64.4% reduction in the total volume of initial US examinations. Under this framework, routine US in children with recurrent UTIs would serve as a crucial clinical safety net to capture potentially missed cases, maintaining a principle similar to current guidelines. Given contemporary healthcare dynamics where early clinical presentation and rapid access to antibiotic therapy are standard, we believe that this selective approach preserves patient safety while substantially mitigating the diagnostic burden on healthcare systems and practitioners.

The incidence of other CAKUT in our study group was also similar to previous reports. The incidence of a DCS without dilation in our cohort was 1.5%, within the expected range of 0.7–4.0% reported in the general population [24–26]. UPJO was identified in 0.2% and UVJO in 0.14% of children, compared to general population rates of 0.2% [27] and 0.07–0.1% [28], respectively. Ureterocele was observed at a rate of 0.2%, versus a reported incidence of 0.025% [25], and unilateral renal agenesis was identified in 0.2% of our cohort, compared to 0.04–0.08% in the general population [29]. These findings, showing a comparable incidence of CAKUT between our study population and the general population, are consistent with the understanding that the underlying cause of the initial UTI in most cases is not attributable to CAKUT.

Notably, follow-up analysis of clinical outcomes in our cohort revealed that VUR was subsequently diagnosed in 34 additional children whose initial urinary tract US showed no abnormalities suggestive of VUR. These diagnoses were made only after further evaluation prompted by recurrent UTIs. This finding highlights an important limitation of US: a normal initial study does not reliably exclude the presence of VUR. Consequently, routine US screening after a first UTI may provide false reassurance while failing to identify children who will later prove to have clinically relevant reflux. Together with the overall low detection rate of CAKUT observed in our study, these data further question the utility of US screening after a first UTI and support a more selective, clinically driven imaging strategy.

Sex-based analysis (univariate and multivariate models) revealed a statistically significant difference in the incidence of UTIs among children older than 6 months, with the condition occurring four to five times more frequently in females than in males, while the incidence of UTI in the children younger than 6 months was similar. Additionally, a statistically significant sex-based difference was identified in the group of children with CAKUT with dilation, which was twice as common in males. Our findings are in line with previous studies that examined sex distribution and concluded that CAKUT with dilation is more frequently observed in males. This difference has been suggested as a possible explanation for the higher incidence of UTIs in males during the first year of life [14, 30, 31].

When comparing all CAKUT cases, urinary tract dilation predominated significantly in both sexes. This finding is consistent with published studies that have concluded that, among all CAKUT, dilation is the most frequently observed abnormality on US [32].

Although US can reveal various anatomical abnormalities, it has limited potential in diagnosing VUR. Firstly, US detects VUR only indirectly through urinary tract dilation. However, other causes for this finding, such as UPJO and UVJO, cannot be reliably differentiated. Secondly, VUR can also be present without urinary tract dilation [9, 17]. Available reports suggest that US has a sensitivity of 16–40% and a negative predictive value of 25–86% for detecting VUR [9]. These rates improve when detecting only high grades of VUR (III-V), which are clinically more important [9]. VCUG is considered the gold standard for diagnosing VUR by the European Association of Urology [33], and US is considered a screening test before performing VCUG in order to avoid unnecessary radiation exposure [9, 17].

Several studies previously evaluated the role of US after the first UTI in children. Shabbeer and Nizamuddin reported US abnormalities in 33.9% of children. In this study, children with signs of UTI were grouped with those with CAKUT, with and without dilation, into a single category of “ultrasound abnormalities” [9]. Subsequent VCUG excluded CAKUT in 46.7% of these cases. Ultimately, CAKUT with dilation was confirmed in 10.6% of the total population, of whom only one-third required further management [9]. Similarly, Jahnukainen et al. found abnormalities in 14.8% of children, with only 2.6% of the total cohort requiring intervention; nevertheless, they concluded that US still has an important role in the management of children with the first UTI [34]. In contrast, Hoberman et al. identified CAKUT with dilation in 12% of children using US, while VCUG performed in the entire cohort revealed VUR in 39% of cases, leading the authors to conclude that US has limited clinical value following the first UTI [10]. Supporting this, Nelson et al. analyzed a group of 2,259 children aged ≤60 months and detected CAKUT with dilation in only 2% of the population, concluding that US is a poor screening test for CAKUT due to its low sensitivity and specificity [8, 16].

Our study has several limitations. First, its retrospective design inherently limits control over data collection and standardization. US examinations were performed by six different pediatric radiologists, which may have introduced interobserver variability in what is already a partially operator-dependent and semi-subjective imaging modality. Second, the timeline for referral to urinary tract US after the first UTI is not clearly determined in the currently accepted national guidelines. As a result, referral patterns have varied among clinicians, affecting the generalizability of our findings. Additionally, the time elapsed between the UTI diagnosis and US examination is not available, which limits additional analysis. Finally, urine cultures were not available for all children included in the study. Consequently, we were unable to analyze potential associations between specific uropathogens and the presence of CAKUT, which may have provided additional clinically relevant insights. Despite these limitations, the large cohort size and real-world clinical setting strengthen the external validity of our findings and provide a meaningful reflection of current practice. Our results, therefore, offer valuable evidence to inform future recommendations and support consideration of a more selective imaging approach after a first UTI in children.

## Conclusion

In this large cohort, routine urinary tract US after a first UTI in children showed a low diagnostic yield, with 92.9% of examinations demonstrating normal anatomy. Clinically significant “CAKUT with dilation” was rare, and the prevalence of VUR was comparable to that of the general population. Notably, additional VUR cases were identified during follow-up in children with initially normal US findings. Our findings suggest that routine US after a first UTI may not represent an optimal use of healthcare resources, given its low diagnostic yield. A two-tier imaging approach may be warranted for infants younger than 6 months and all male children following a first UTI, and all children with recurrent UTIs. This selective, risk-based approach may optimize resource use while maintaining clinical safety.

## Data Availability

The data that support the findings of this study are not openly available due to reasons of sensitivity and are available from the corresponding author upon reasonable request. Data are located in controlled access data storage at University Medical Centre Ljubljana
